# Topical Application of VitB6 Ameliorates PM2.5-Induced Dry Eye via NFκB Pathway in a Murine Model

**DOI:** 10.3390/biomedicines13030541

**Published:** 2025-02-21

**Authors:** Jinyu Hu, Yanmei Zeng, Liying Tang, Lei Ye, Cheng Chen, Qian Ling, Xiaoyu Wang, Liangqi He, Xu Chen, Yixin Wang, Qianmin Ge, Yi Shao

**Affiliations:** 1 Department of Ophthalmology, Shanghai General Hospital, National Clinical Research Center for Eye Diseases, Shanghai Jiao Tong University School of Medicine, Shanghai 200080, China; hujinyuu@outlook.com (J.H.); blossomay2095@163.com (Y.Z.); yelei10062025@163.com (L.Y.); cc_sjcd@163.com (C.C.); 15797864223@163.com (X.W.);; 2 Department of Ophthalmology, The First Affiliated Hospital of Nanchang University, Nanchang 330006, China; 3 Department of Ophthalmology, Zhongshan Hospital of Xiamen University, Xiamen 361102, China; tangly0817@sina.com; 4 Ophthalmology Centre, Maastricht University, 6200 MS Maastricht, Limburg Provincie, The Netherlands; 5 School of Optometry and Vision Sciences, Cardiff University, Cardiff CF24 4HQ, UK

**Keywords:** dry eye, particulate matter 2.5 (PM2.5), inflammation, VitB6, NF-κB pathway

## Abstract

**Background/Objectives:** Dry eye (DE) is mainly characterized by dryness, foreign body sensation, eye pain and visual impairment. Their possible causes are mainly inflammation, tissue damage and neurosensory abnormalities, and vitamin B6 (VitB6) attenuates the inflammatory response by modulating the NF-κB pathway to quench reactive oxygen species (ROS). The aim of this experiment was to investigate the therapeutic effect of VitB6 eye drops on particulate matter 2.5 (PM2.5)-induced dry eye in mice. **Methods**: Mice induced with the dry eye group were first induced using PM2.5 eye drops in a standard environment for 14 days, and then treated with different concentrations of VitB6 eye drops for 14 consecutive days. The phenol red cotton test was used to measure tear production. Ocular inflammation index and tear film function were evaluated by slim microscopy. Hematoxylin–eosin (HE) staining was used to observe conjunctival and corneal structure. Periodate–Schiff (PAS) staining was used to quantify conjunctival goblet cells. Corneal cell apoptosis was determined by TUNEL assay. The expression of keratin 10 (K10) and p-NF-κB p65 was detected by immunofluorescent staining and Western blot analysis. **Results**: Mice using only the PM2.5 model all exhibited varying degrees of dry eye symptoms. VitB6 treatment increased tear secretion and reduced inflammatory indices in mice with increased nerve density and number of branches in the basement membrane of the corneal epithelium. **Conclusions**: We found that administering VitB6 eye drops has a therapeutic effect in PM2.5-induced DE. This observation suggests that VitB6 may be useful in the clinical therapy of DE.

## 1. Introduction

Dry eye (DE) is a multifactorial disease of the ocular surface characterized by a loss of tear film homeostasis and associated ocular symptoms. It is a widespread and common eye disease that exists worldwide. Epidemiological studies show that air pollution, wind, low humidity and high altitude can lead to an increased incidence of DE [1]. Dryness, redness, itching, and severe pain in the eyes are its main symptoms and it can progress further to corneal ulcers, loss of vision, and even blindness. The causes of DE include tear film instability, tear hyperosmolarity, ocular surface inflammation and injury, and neurosensory abnormalities [2]. Chronic DE can lead to corneal nerve damage and corneal intuition loss. In addition to affecting eye function, DE affects daily activities, the psychology and work of the patient, and consequently reduces the quality of life of the population. There are many risk factors for DE, which are mainly age, use of electronic devices, wear and tear of corneal contact lenses, systemic diseases, and especially the influence of environmental factors [3,4].

The impact of air pollution on human health is a hot topic of global concern, and the problem of air pollution is still very serious in low-income countries [5]. PM2.5 is defined as a harmful type of air fine particulate matter with a diameter of less than 2.5 μm. PM2.5 mainly comes from burning fuels, such as motor vehicle emissions and coal combustion. PM2.5 has an extremely complex composition, including a large number of organic substances (e.g., benzo (a) pyrene and polycyclic aromatic hydrocarbons), as well as numerous inorganic components, such as sulfates, nitrates, and heavy metals [6]. Current research has confirmed that PM2.5 is harmful to both the respiratory [7] and cardiovascular systems [8], and as early as 2009, a study in the USA reported that PM2.5 also shortens the average life expectancy of individuals [9]. For exposed eye surfaces, PM2.5 has an even more direct impact. A growing number of studies have shown that air pollution can cause DE [10]. Most of the modalities currently used for DE treatment are symptomatic in nature and all have certain drawbacks. For example, most tear substitutes contain preservatives, electrolytes, and buffers that affect the homeostasis of the tear film to varying degrees, and they are mostly used in patients with mild to moderate DE. Glucocorticoids and non-steroidal anti-inflammatory drugs can significantly reduce inflammation and relieve ocular discomfort, but complications associated with their use cannot be ignored. Immunomodulators and some biologics are effective but need further evaluation in clinical trials. A surgical approach has clear indications and contraindications and is mainly used in severe cases, most of which cannot be reversed. Therefore, it is urgent to find an ideal drug for the treatment of air pollution as a common predisposing factor.

Vitamin B6 (VitB6), also known as pyridoxine, includes three naturally occurring forms, pyridoxal, pyridoxine and pyridoxamine, and is a water-soluble vitamin that exists in the body as a phosphate ester. VitB6 plays a coenzyme role in various biochemical reactions and is mainly involved in the metabolism of amino acids, nucleic acids, glycogen and lipids [11]. Since its discovery in the 1930s, in addition to being an essential nutrient for the body, VitB6 has been considered an antioxidant and anti-inflammatory agent [12] and has been used to regulate immunity and gene expression [13]. It has been shown that VitB6 quenches reactive oxygen species (ROS) and attenuates the inflammatory response, mainly through the regulation of the NF-κB pathway [14,15]. Current studies have also demonstrated that B vitamins have a significant role in promoting nerve repair and alleviating neurological dysfunction [16,17]. Given the pathogenesis of dry eye, it remains to be investigated whether VitB6 is equally effective in the treatment of dry eye.

Accordingly, we hypothesized that topical application of VitB6 eye drops would suppress effectively the ocular surface inflammation in dry eye. Thus, we investigated the effect of topical application of VitB6 eye drops on epithelial damage in the ocular surface using a murine dry eye group induced by PM2.5.

## 2. Materials and Methods

### 2.1. Laboratory Animal Preparation

Sixty healthy male Balb/c mice, 6–8 weeks old, that were special pathogen free (SPF), weighing 18–21 g, were provided by the Experimental Animal Centre of Xi’an Jiaotong University School of Medicine (Xi’an, China). All mice underwent fundus examination prior to the experiment. The mice were anesthetized by intranasal inhalation of 1% isoflurane gas (RWD Life Science Co., Shenzhen, China). The pupils were dilated by ordering eye drops containing a mixture of 0.5% tropicamide (Wuhan Wujing Pharmaceutical Co., Wuhan, China) and 0.5% phenylephrine hydrochloride (Shanghai Harvest Pharmaceutical Co., Shanghai, China). The fundus of mice was observed using SD-OCT. There were no abnormalities in the fundus of all mice. SIT results were ≥10 mm/5 min. Throughout the study, mice were housed in a standard environment: room temperature 25 °C ± 1 °C, humidity 60% ± 10% and alternating 12 h light and dark cycles (AM 8:00 to PM 8:00) and given the same water and chow. All experimental methods involved in this study followed the Declaration of Helsinki and animal experiments were performed in accordance with the Association for Research in Vision and Ophthalmology (ARVO) regulations for the use of laboratory animals in ophthalmic research and were approved by the Animal Ethics Committee of the School of Medicine, Xi’an Jiaotong University (date of issue: 16 September 2016; expiry of the approval: five years; the approval number: 20160916).

### 2.2. PM2.5 Collection and Preparation of Eye Drops

PM2.5 was obtained from a super station of the Xi’an Municipal Environmental Monitoring Station (sampling was conducted continuously for 22 h a day from 10:30 am to 8:30 am the next day). Sampling was carried out using a TH-16A four-channel intelligent sampler for atmospheric particulate matter (Wuhan Tianhong Instrument Ltd., Wuhan, China) with a cut particle size of 2.5 μm and filtered with Whatman Polytetrafluoroethylene (PTFE) membranes [18,19]. For sampling, the PTFE membranes containing PM2.5 were cut to 1 cm × 1 cm size, immersed in deionized water, and shaken three times with ultrasound for 45 min each time; then the membranes were filtered with six layers of gauze, and the washed PM2.5 suspension was freeze-dried under vacuum, weighed, stored in a refrigerator at 4 °C and set aside. For the preparation of PM2.5 eye drops, PM2.5 samples were diluted in sterile PBS to form a concentration of 5 mg/mL and then were vortexed ultrasonically. The preservative benzyl bromide was added to two groups of eye drops (PM2.5 and phosphate-buffered saline [PBS]) with the concentration controlled at 0.005%. The eye drops were kept at 4 °C.

### 2.3. Model Making and Grouping

Sixty healthy male Balb/c mice were housed in a standard environment. Ten were randomly selected as normal controls, and the remaining fifty mice were dosed with 5 mg/mL PM2.5 eye drops (all right eyes were selected) to induce a dry eye mouse model [19]. The PM2.5 dosage was 5 μL each time, 4 times a day for 14 days. Because the conjunctival sacs of Balb/c mice are small, this experiment was performed with a micropipette gun, and the eye dosing was performed in a non-anesthetized state by grasping the mice with bare hands, and the eye dosing was performed by an assistant, and each operation was performed by the same operator. Screening of all clinical indicators in 50 mice after 14 days. Screening criteria [19]: tear film breakup time (TBUT) ≤ 4 s. Screening criteria: total score > 8. A total of 40 mice (right eye) that met the criteria for dry eye were screened by measuring all indexes and randomly divided into four groups: dry eye group, PBS control group, 0.02% VitB6 (Alfa Aesar Chemical Co., China) treatment group, and 0.05% VitB6 treatment group: Group A (dry eye group, n = 10, right eye, no treatment), Group B (PBS treatment group, n = 10, right eye, 4 times/day), Group C (0.02% VitB6 treatment group, n = 10, right eye, 4 times/day), and Group D (0.05% VitB6 treatment group, n = 10, right eye, 4 times/day). On days 0, 7 and 14 of the experiment, Schirmer I test (SIT), TBUT, fluorescein (FL), Rose bengal staining (RB), Lissamine Green staining (LG), inflammatory index scores and confocal microscopy were performed in each group by time. The mice were executed by 2% chloral hydrate (0.1 mL/10 g) on the 14th day of treatment, and the eye tissues were taken for histological analysis and Western blot (WB) assay.

### 2.4. SIT

Phenol red cotton thread (FCI Ophthalmics, Pembrooke, MA, USA) was taken under standard conditions at the same time point (PM 3:00) on (treatment days 0, 7 and 14), and the length of the reddened portion of the thread was measured using vernier calipers and repeated three times for each eye, the average was taken as the final length and the data recorded. The mouse eyelids were gently closed artificially after the test to avoid corneal exposure and ocular surface irritation. Screening criteria [19]: the length of the phenol red cotton thread was 1.8 ± 0.3 mm.

### 2.5. TBUT Test

At the same time point (3 pm) on treatment days 0, 7 and 14 in a standard environment, a drop of 0.1% sodium fluorescein solution (Jingming New Technology Development Co., Tianjin, China) was applied to the surface of the right eye of the mice, which was artificially transient 3 times and immediately observed under a slit lamp microscope (Zeiss, Jena, Germany) with cobalt blue light (40×), and the time taken for the first tear film rupture point to appear in the stained area was recorded. This was repeated 3 times, and the average was taken and recorded. Screening criteria [19]: TBUT ≤ 4 s.

### 2.6. FL Staining

At the same time point (3 pm) on treatment days 0, 7 and 14 in a standard environment, a 0.1% sodium fluorescein solution was dropped into the conjunctival sac of the right eye of the mice after which they were artificially made to transiently look at the cornea 3 times, and after 90 s, the corneal FL staining was observed under cobalt blue light (40×) in a slit lamp microscope. The corneal staining points were scored (total score 16 points). Screening criteria [19]: total score 8–12. Scoring method for FL staining: 0 points: no staining at all; 1 point: small amount of punctate staining but less than 30 points; 2 points: punctate staining over 30 points but no spread; 3 points: diffuse punctate staining but no plaques; 4 points: plaque staining. We used the same person at the same time, place and setting for each examination.

### 2.7. LG Staining and RB Staining

At the same time point (3 pm) on treatment day 0, 7 and 14 in a standard environment, after staining the corneas of mice with LG test strips (Jingming New Technology Development Co., Ltd., Tianjin, China), the mice were made to transiently look 3 times and then observed under a slit lamp microscope (40×), photographed, and the corneal staining was recorded (screening criteria [20]: total score of 5–7). Screening criteria: 0 score: no staining; 1 score: corneal staining is less than one-third; 2 points: corneal staining up to two-thirds; 3 points: more than two-thirds staining. After completing the observation of LG staining, mice were stained with Tiger Red, and a drop of 1% Tiger Red solution (Sigma-Aldrich, St. Louis, MO, USA) was added to the conjunctival sac of the mice and scored 15 s later under a slit lamp microscope (40×) using the Van Bijsterveld three-divisional grading method. Screening criteria: The entire ocular surface was divided into three regions: (A) conjunctival of the nasal palpebral fissure, (B) conjunctival of the temporal fissure, and (C) cornea. Each zone is scored out of 3 points, and the scores for each zone are then added together to obtain the total score (9 points). Each inspection should be carried out by the same person at the same time, place, and under the same lighting intensity, humidity, and temperature. Finally, the cornea and conjunctiva were observed and photographed under the slit lamp microscope, and the staining was recorded: 0 score: no staining; 1 point: a little spot staining; 2 points: somewhere in between; 3 points: fused into a piece of staining.

### 2.8. Evaluation of Inflammation

The corneal conditions including edema, transparency and neovascularization were assessed by slit lamp microscopy on days 0, 7 and 14 of treatment, and the inflammatory indices were evaluated as previously described [21]. Briefly, the inflammatory index was calculated as the sum of the scores of the following parameters divided by 9: the thick- ness of the ciliary hyperemia (0: absent; 1: less than 1 mm; 2: 1 to 2 mm; 3: more than 2 mm); the presence of central corneal edema (0: absent; 1: present with visible iris details; 2: present without visible iris details; 3: present without visible pupil); and the presence of the peripheral corneal edema (0: absent; 1: present with visible iris details; 2: present without visible iris details; 3: present with no visible iris).

### 2.9. Confocal Microscopy

The distribution of subbasal nerve fibers in the corneal epithelium of each group of mice was observed using a Confoscan4 slit-scanning confocal microscope (NidekCi, Kyoto, Japan). AUTOCAD software (Version: AutoCAD 2016, Auto Desk Co., SAN Rafael, CA, USA) was applied to determine the length of the subbasal nerve fibers in the cornea. The ACCMetrics software (Version: ACCMetrics V.2) was used to count the total number of branches of nerve fibers in each image. Curvature score calculation: The degree of curvature of the nerve fibers in the images was divided into 4 levels. The higher the score, the greater the nerve fiber curvature [22].

### 2.10. Ocular Tissue Sampling

Mice were anesthetized with 10% chloral hydrate, and ocular tissues, including eyelids, conjunctiva and eyeball (including cornea), were removed. The ocular tissues were fixed in 4% paraformaldehyde solution (Dingguo Biotechnology Co., Beijing, China) for 24 h. In addition, the corneal tissues from the WB test were quickly placed in a freezing tube and stored in an ultra-low temperature refrigerator (Thermo, Waltham, MA, USA) at −80 °C.

### 2.11. Hematoxylin–Eosin (HE) Staining and Periodic Acid–Schiff (PAS) Staining

The cryopreserved ocular tissue was paraffin sectioned to a thickness of 4 μm, then stained with HE and PAS and sealed with neutral gum, placed under a light microscope (Carl Zeiss AG, Oberkochen, Baden-Wurttemberg, Germany) to observe the staining, photographed and recorded. Goblet cell density was determined by counting PAS-positive cells in four different sections of each animal and taking the average [19,23].

### 2.12. Corneal TUNEL (Terminal Deoxynucleotidyl Transferase-Mediated dUTP Nick End Labeling) Staining

Corneal tissues were taken after 14 days of VitB6 or PBS treatment and stained for apoptosis detection using terminal deoxynucleotidyl transferase-mediated cut-end labeling (TUNEL kit). Nuclei were re-stained with DAPI, and the staining results were observed under a fluorescent microscope (OLYMPUS, Westburg, NY, USA) and photographed.

### 2.13. Immunofluorescence Staining

After sections of ocular tissue were frozen, the specimens were treated with PBS buffer and then dehydrated, embedded (OCT embedding agent) and stored at −80 °C. The tissue was sectioned (thickness 5 μm), mounted on slides, soaked in PBS buffer for 10 min, permeabilized with 0.2% TritonX-100 (Invitrogen, Carlsbad, CA, USA) for 10 min, washed 3 times in PBS buffer, and closed for 1 h at room temperature in 1% BSA wet boxes. Rabbit anti-mouse Keratin 10 (K10) monoclonal antibody (Abcam, Boston, MA, USA) was used at a 1:200 dilution as the primary antibody, and the diluted primary antibody (K10, ratio 1:200) was added to PBS buffer, at 4 °C. After overnight treatment with PBS buffer, the nuclei were re-stained with DAPI and washed with PBS buffer, and finally fluorescence blocked, observed under fluorescence microscope and photographed.

### 2.14. WB

Corneal tissue was weighed, lysate was added, ground, centrifuged in an ultra-low-temperature centrifuge (12,000 rpm × 10 min) and the supernatant was removed. Protein quantification (BCA protein quantification kit, Thermo, USA) was performed, and the samples were subjected to SDS-PAGE electrophoresis to separate the proteins. After electrophoresis, the gel was cut and the membrane was transferred. After the transfer of the membrane, the PVDF membrane (Milliporer, Burlington, MA, USA) was stained in Rejuveno Red staining solution for 2–5 min to observe the effect of protein transfer. We used 5% skimmed milk to seal the polyvinylidene fluoride (PVDF) membrane and the primary antibody dilution overnight at 4 °C. Rabbit anti-mouse NF-κB p65 polyclonal antibody (Abcam, USA) was used at a 1:1000 dilution and Rabbit anti-mouse phospho-NF-κB p65 polyclonal antibody (Abcam, USA) was used at a 1:800 dilution. The next day, the membranes were washed with TBST (TBS with Tween-20) buffer for 10 min × 3 times and then placed in secondary antibody dilution solution (Goat Anti-Rabbit Horseradish Peroxidase-Labeled Antibody (Zhongshan Jinqiao Co., Zhongshan, China) was used at a 1:10,000 dilution) and incubated on a decolorized shaker with slow shaking for 1 h. The membranes were washed with TBST for 10 min × 3 times and then incubated with a Bio-Rad gel Developer (Bio RAD, Hercules, CA, USA) exposure, and the protein bands were quantified in gray scale using β-actin (mouse anti-rat β-actin monoclonal antibody [Abcam, USA]) as a reference, and the experiment was repeated 3 times.

### 2.15. Statistical Analysis

AUTOCAD software was used to analyze the distribution of corneal nerve fibers, and Image J image analysis software (Version 1.53) was used to process the immunofluorescence staining images and WB images. SPSS 22.0 software was used to analyze the data. Analysis of variance with repeated measures design was used to compare values between groups and at each time point. The LSD-t test was used to compare the differences between the two groups at each time point. Differences were considered statistically significant at *p* < 0.05.

## 3. Results

### 3.1. Topical Application of VitB6 Alleviated PM2.5-Induced Ocular Surface Damage

The study included a DE group, PBS control group, 0.02% VitB6-treated group, and 0.05% VitB6-treated group. There were no significant differences in SIT (phenol red cotton thread length), TBUT, FL, RB, LG, and inflammation index scores among the four groups of mice before treatment (*p* > 0.05, Table 1, Table 2 and Table 3). The comparison of SIT, TBUT, FL, RB, LG, and inflammation index scores among the four groups of mice after days 7 and 14 showed that the treatment effect became more pronounced with the passage of time: (1) SIT, TBUT, FL, RB, LG, and the inflammation index at different times were F = 112.555 (*p* = 0.0001), F = 71.245 (*p* = 0.0001), F = 14.491 (*p* = 0.0001), F = 46.385 (*p* = 0.0001), F = 40.009 (*p* = 0.0001), and F = 17.526 (*p* = 0.0001), respectively. (2) There were differences in SIT, TBUT, FL, LG, and the inflammatory index among the four groups: F = 17.857 (*p* = 0.0001), F = 6.247 (*p* = 0.002), F = 9.306 (*p* = 0.0001), F = 11.483 (*p* = 0.0001), and F = 7.805 (*p* = 0.0001), respectively. There was no significant difference in RB scores among the four groups (F = 2.481; *p* = 0.077). (3) There were differences in the trends of SIT, TBUT, FL, RB, LG, and the inflammation index among the four groups: F = 167.453 (*p* = 0.0001), F = 126.790 (*p* = 0.0001), F = 23.516 (*p* = 0.0001), F = 76.842 (*p* = 0.0001), F = 93.452 (*p* = 0.0001), and F = 27.238 (*p* = 0.0001), respectively (Table 1, Table 2 and Table 3).

**Figure 1:**
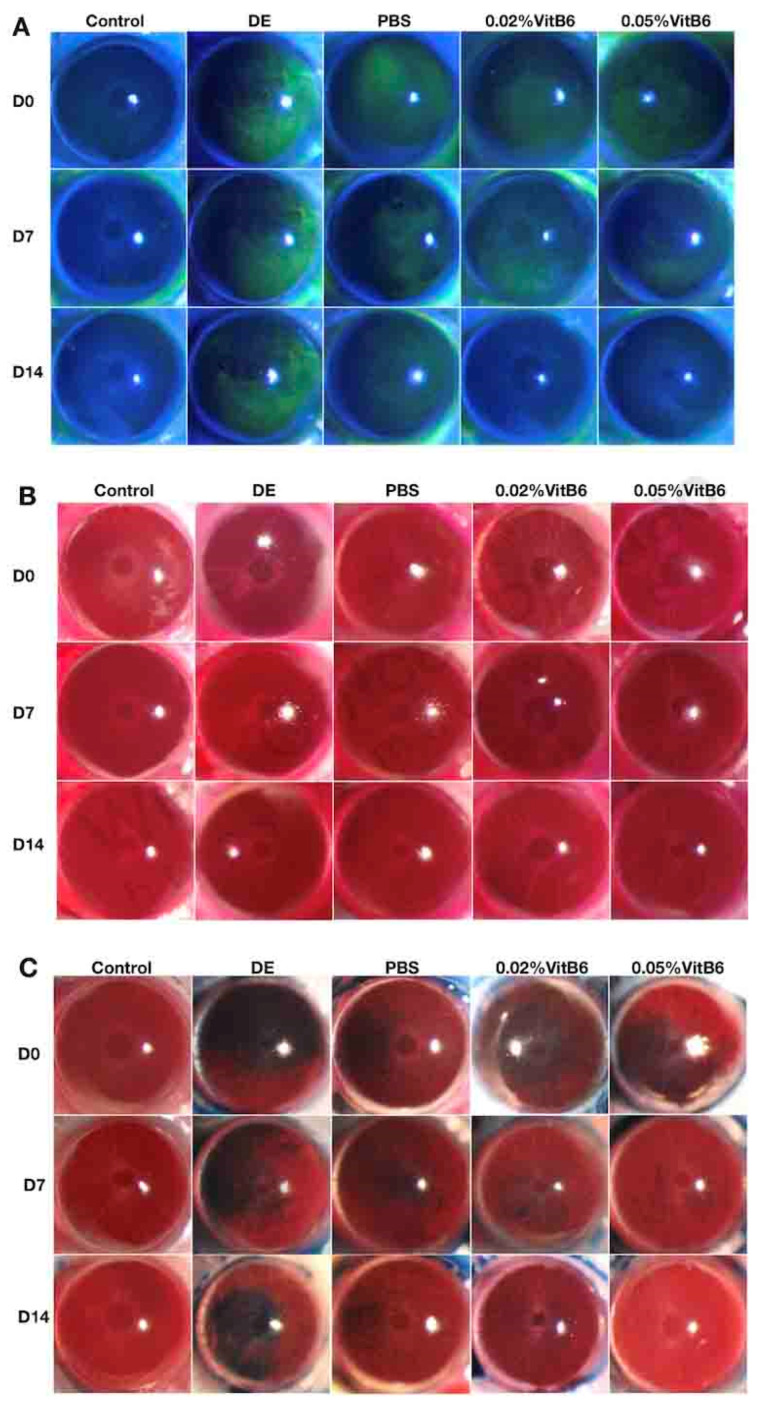
Representative pictures of the changes in the ocular surface and the degree of inflammation in different groups of mice before and after dry eye treatment. (**A**) FL staining. At the beginning of the experiment, the DE, PBS, 0.02%, and 0.05% groups showed patchy staining of corneal FL, and after 14 days of treatment, the 0.02% and 0.05% groups showed a significant decrease in corneal staining. (**B**) RB staining. At the beginning of the experiment, the DE group, PBS group, 0.02% group and 0.05% group showed large patchy staining of corneal RB staining, and after 14 days of treatment, the 0.02% group and 0.05% group showed a significant decrease in corneal staining. (**C**) LG staining. At the beginning of the experiment, the DE group, PBS group, 0.02% group and 0.05% group showed large staining of corneal LG staining, and after 14 days of treatment, the 0.05% group showed almost disappearance of corneal staining. Slit lamp (40×). Image taken by EOS 200D (Canon, Tokyo, Japan). (n = 10 mice/group). Abbreviations: D, Day; DE, dry eye; PBS, phosphate-buffered saline; VitB6, vitamin B6; FL, fluorescein; RB, Rose bengal; LG, Lissamine Green.

**Figure 2:**
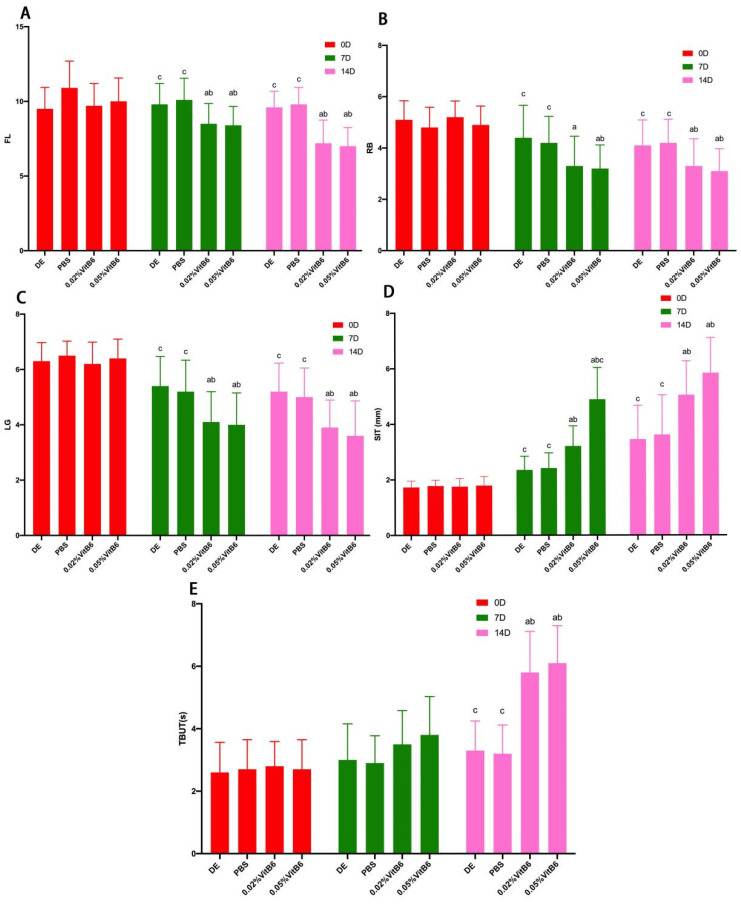
Comparative analysis graphs of different test results in dry eyes. (**A**) FL staining results before treatment, on the 7th day of treatment, and on the 14th day of treatment. The corneal staining was reduced in the 0.02% VitB6 treatment group and the 0.05% VitB6 treatment group starting from the 7th day of treatment. (**B**) RB staining results before treatment, on day 7 of treatment, and on day 14 of treatment. Corneal staining was reduced in the 0.02% VitB6 treatment group relative to the DE group at day 7; corneal staining was reduced in the 0.05% VitB6 treatment group relative to the DE and PBS treatment groups. At day 14 of treatment, corneal staining was reduced in the 0.02% VitB6 treatment group and the 0.05% VitB6 treatment group. (**C**) LG staining results before treatment, on day 7 of treatment, and on day 14 of treatment. Corneal staining was reduced in the 0.02% VitB6-treated group and the 0.05% VitB6-treated group starting from the 7th day of treatment. (**D**) SIT results before treatment, on day 7 of treatment, and on day 14 of treatment. Tear secretion increased in both the 0.02% VitB6-treated group and the 0.05% VitB6-treated group starting on day 7 of treatment, and the increase was statistically significant in the 0.05% VitB6-treated group relative to the 0.02% VitB6-treated group by day 7. (**E**) TBUT test results before treatment, on day 7 of treatment, and on day 14 of treatment. At day 7 of treatment, there was no significant difference in BUT assay results between the four groups. On day 14 of treatment, the BUT time was significantly prolonged in both the 0.02% VitB6 treatment group and the 0.05% VitB6 treatment group. Note: ^a^ *p* < 0.05, vs. DE; ^b^ *p* < 0.05, vs. PBS; ^c^ *p* < 0.05, vs. 0.02% VitB6. Abbreviations: D, Day; DE, dry eye; PBS, phosphate-buffered saline; VitB6, vitamin B6; FL, fluorescein staining; RB, Rose bengal staining; LG, Lissamine Green staining; SIT, Schirmer I test; TBUT, Tear break-up time.

After 14 days of treatment, corneal FL, RB, and LG scores were statistically lower in the 0.02% VitB6 and 0.05% VitB6 treatment groups compared to the DE model group and the PBS treatment group: F = 14.167 (*p* = 0.0001), F = 3.673 (*p* = 0.021), F = 5.280 (*p* = 0.004; Figure 1A–C and Figure 2A–C, Table 2 and Table 3). There was also some improvement in the corneal inflammation index with increased length of the phenol red cotton threads and longer TBUT: F = 7.966 (*p* = 0.0001), F = 19.928 (*p* = 0.0001), and F = 14.542 (*p* = 0.0001; Figure 2D,E, Table 1 and Table 2). Although no statistical difference was found between the two concentrations of VitB6 eye drops, i.e., 0.02% and 0.05%, the treatment trend for a number of indicators showed that the latter was more effective than the former (Figure 2).

### 3.2. Topical Application of VitB6 Alleviated Corneal Stromal Nerve Fibers of PM2.5-Induced Dry Eye in Mice

**Figure 3:**
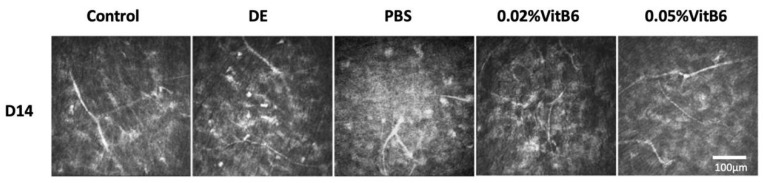
Representative pictures of nerve fibers in different groups of mice after 14 days of treatment. The distribution of nerves under the basement membrane of corneal epithelium was observed under confocal microscope after 14 days of treatment, and the corneal stromal nerve fibers were clear and straight in the control group. In the DE group, PBS treatment group, 0.02% VitB6 and 0.05% VitB6 treatment groups, the nerve fibers in the corneal stroma were curved, while the curvature of the nerve fibers in the 0.02% VitB6 and 0.05% VitB6 groups improved and the nerve fiber density increased (n = 10 mice/group). Scale bars: 100 µm. Abbreviations: D, Day; DE, dry eye; PBS, phosphate-buffered saline; VitB6, vitamin B6.

Using confocal microscopy, the corneal stromal nerve fibers were clear and straight in the normal control group and curved in the remaining four groups, while the curvature of the nerve fibers improved in the 0.02% VitB6 and 0.05% VitB6 groups (Figure 3).

Comparison of nerve fiber density, branch count, and curvature scores among the four groups before treatment, at 7 days, and at 14 days of treatment: (1) There were differences in the nerve fiber density and branch count at different times: F = 8.400 (*p* = 0.001) and F = 8.750 (*p* = 0.000), respectively. There was no significant difference in curvature scores among the times (F = 2.323, *p* = 0.114). (2) There was a difference in nerve fiber density and the number of branches among the four groups: F = 4.976 (*p* = 0.005) and F = 5.734 (*p* = 0.003), respectively. There was no significant difference in curvature scores among the four groups (F = 2.173, *p* = 0.108). The trend in nerve fiber density and branch count differed among the four groups: F = 13.172 (*p* = 0.001) and F = 15.411 (*p* = 0.0001), while the trend in curvature scores did not differ significantly among the four groups (F = 3.748, *p* = 0.061; Table 4, Table 5 and Table 6).

By measuring the results for nerve fiber density, branch numbers, and curvature scores before and after treatments in each group, it was found that the differences between the pre-treatment DE model group, PBS treatment group, 0.02% VitB6 group, and 0.05% VitB6 treatment group were not statistically significant: F = 0.029 (*p* = 0.993), F = 0.467 (*p* = 0.707), and F = 0.026 (*p* = 0.994), respectively. After 7 days of treatment, there was a difference in nerve fiber density and branch numbers among the four groups: F = 3.809 (*p* = 0.018) and F = 3.273 (*p* = 0.032). No significant difference was seen in the curvature scores (F = 0.704, *p* = 0.056). After 14 days of treatment, there were differences in the nerve fiber densities, number of branches, and curvature scores among the four groups: F = 6.092 (*p* = 0.002), F = 8.434 (*p* = 0.0001), and F = 4.113 (*p* = 0.013, respectively; Table 4, Table 5 and Table 6). The density, number of branches, and curvature scores of the corneal subepithelial nerves in the 0.02% VitB6 and 0.05% VitB6 groups were better than those of the PBS and DE model groups.

### 3.3. Topical Application of VitB6 Improved Morphology of Corneal and Conjunctiva Epithelial Cells of PM2.5-Induced Dry Eye in Mice

**Figure 4:**
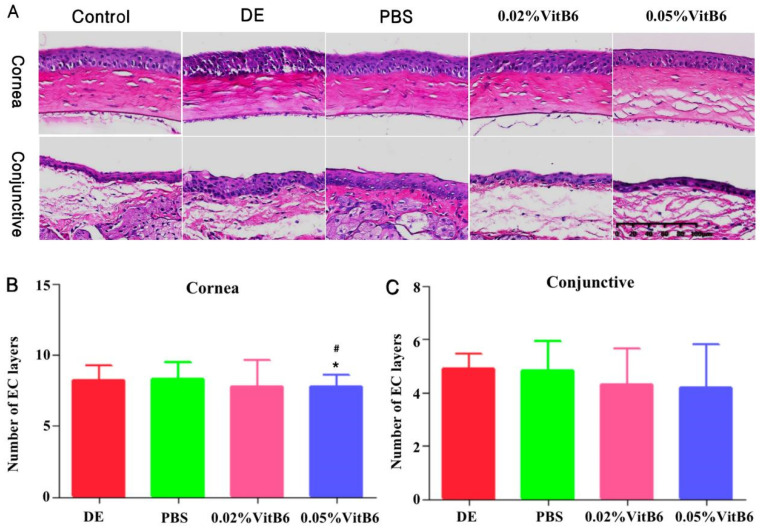
Representative pictures of HE staining of the corneal conjunctiva of mice in different groups after 14 days of treatment. (**A**) The central corneal and conjunctival epithelial cells in the DE and PBS-treated groups were disorganized, with thickened layers and inflammatory cell infiltration in the stroma. In contrast, the central corneal and conjunctival epithelium of 0.02% VitB6 and 0.05% VitB6 treatment groups were smooth and the cell morphology gradually normalized. (**B**) Comparison of changes in the number of layers of epithelial cells after 14 days of treatment with two different concentrations of VitB6 eye drops. (**C**) No significant difference in the number of layers of conjunctival epithelial cell layers was observed in the four groups of mice. Note: * *p* < 0.05, vs. dry eye, # *p* < 0.05, vs. PBS. (n = 10 mice/group). Scale bars: 20 µm. Abbreviations: DE, dry eye; PBS, phosphate-buffered saline; VitB6, vitamin B6.

The HE staining results showed that, after 14 days of treatment, the central corneal and conjunctival epithelial cells in the DE model group and the PBS-treated group were disorganized, and they had thickened layers and inflammatory cell infiltration in the stroma. In contrast, the central corneal and conjunctival epithelium of the 0.02% VitB6- and 0.05% VitB6-treated groups were smooth and the cell morphology gradually normalized (Figure 4A). In comparison to the other two groups, there was a statistically significant difference in the number of corneal epithelial layers 14 days following the effect of two different amounts of VitB6 drops (F = 3.605, *p* = 0.014; Figure 4B); however, no significant difference was seen in the number of layers of conjunctival epithelial cells (F = 1.264, *p* = 0.288; Figure 4C).

### 3.4. Topical Application of VitB6 Suppressed PM2.5-Induced Apoptosis in Ocular Surface

**Figure 5:**
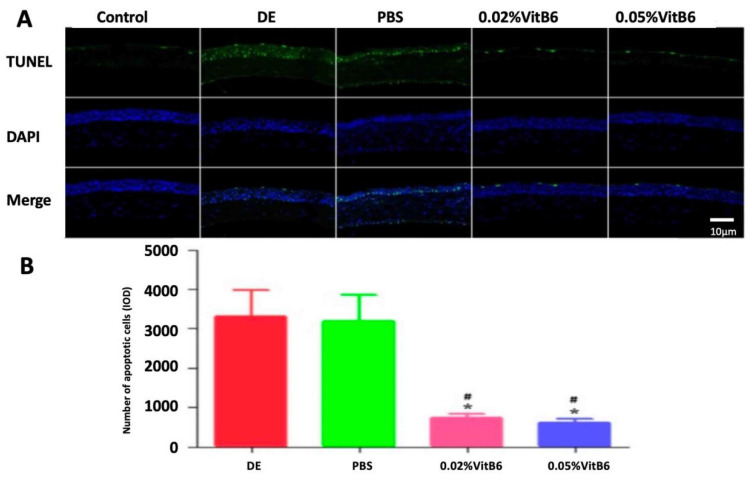
Comparison of TUNEL staining results of different groups of mice after 14 days of treatment. (**A**) Corneal sections of mice were stained with TUNEL and then nuclei (blue) were re-stained with DAPI, and the number of apoptotic cells was indicated in green. The number of apoptotic cells in the control group was minimal, and after 14 days of treatment, the number of apoptotic cells in the 0.02% VitB6 and 0.05% VitB6 treatment groups was reduced compared with the dry eye group and the PBS treatment group. (**B**) The IOD value was used to determine the number of TUNEL-positive cells in the central cornea of mice, and the IOD value in the 0.02% VitB6 and 0.05% VitB6 treatment groups was lower than the dry eye group and the PBS treatment group. (n = 10 mice/group). Scale bars: 10 µm. Note: * *p* < 0.05, vs. dry eye, # *p* < 0.05, vs. PBS. Abbreviations: DE, dry eye; PBS, phosphate-buffered saline; VitB6, vitamin B6; TUNEL, terminal deoxynucleotidyl transferase-mediated end-of-cut labeling.

The TUNEL staining results showed minimal apoptosis in the normal control group, and after 14 days of treatment, the number of apoptotic cells was reduced in the 0.02% VitB6- and 0.05% VitB6-treated groups compared to the DE model group and PBS-treated group (Figure 5A). The IOD values of TUNEL-positive cell count in the central cornea of mice were evaluated and were significantly lower in the 0.02% VitB6- and 0.05% VitB6-treated groups than in the dry eye and PBS-treated groups after 14 days of treatment (F = 94.86, *p* = 0.0001; Figure 5B).

### 3.5. Topical Application of VitB6 Improved Global Cells of PM2.5-Induced Dry Eye in Mice

**Figure 6:**
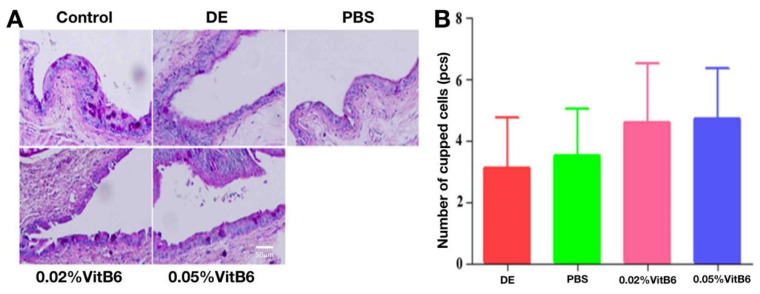
Comparison of PAS staining of the domed conjunctiva in different groups of mice after 14 days of treatment. (**A**,**B**) There was no significant difference in the number of conjunctival cupped cells in different groups of mice (n = 10 mice/group). Scale bars: 50 µm. Abbreviations: DE, dry eye; PBS, phosphate-buffered saline; VitB6, vitamin B6; PAS, Periodic acid–Schiff.

The integrity of the ocular surface tear film is aided by conjunctival cupping cells. The number of conjunctival cupped cells decreased in the dry eye model group and the PBS-treated group of mice after 14 days of treatment (Figure 6A), but there was no significant difference in the number of conjunctival cupped cells between the two groups of mice after VitB6 eye drops (F = 2.590, *p* = 0.068) in the current experiment (Figure 6B).

### 3.6. Topical Application of VitB6 Alleviated PM2.5-Induced Keratin in Ocular Surface

**Figure 7:**
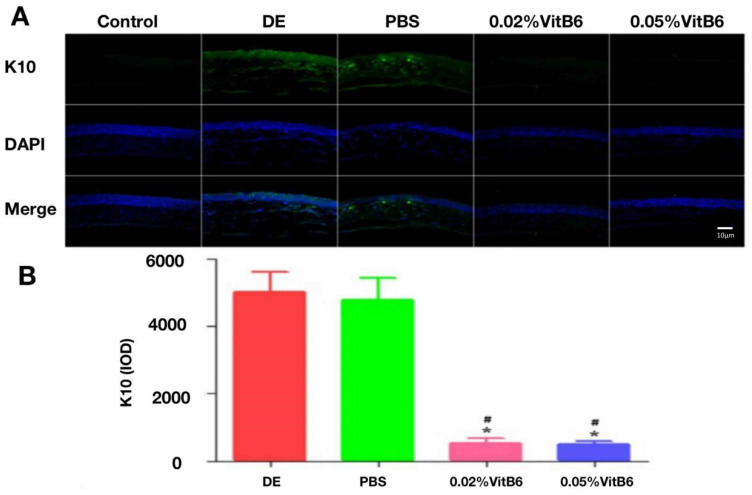
Comparison of corneal K10 expression in different groups of mice after 14 days of treatment. (**A**) The nuclei (blue) were re-stained with DAPI after immunofluorescence staining for K10, and the expression of K10 protein in the central cornea was highlighted in green. Control group mice did not express K10, and the central corneal expression of K10 in mice treated with VitB6 for 14 days was lower than in the dry eye group and the PBS-treated group. (**B**) The IOD values of K10 in the central cornea of mice treated with 0.02 percent VitB6 and 0.05 percent VitB6 were considerably lower than those in the PBS treatment group and the dry eye group after 14 days of therapy (n = 10 mice/group). Scale bars: 10 µm. Note: * *p* < 0.05, vs. dry eye, # *p* < 0.05, vs. PBS. Abbreviations: DE, dry eye; PBS, phosphate-buffered saline; VitB6, vitamin B6; K10, keratin 10.

Normal control animals did not express keratin 10 (K10); however, K10 expression in the central cornea of mice treated with VitB6 for 14 days was lower than in the DE model and PBS-treated groups (Figure 7A). The number of corneal K10-positive cells in the four groups was assessed for IOD values after 14 days of treatment, and the results revealed that the IOD values of central corneal K10 in the 0.02 percent VitB6 and 0.05 percent VitB6-treated groups were significantly lower than the PBS-treated group and the DE group, with statistically significant differences (F = 315.38, *p* = 0.0001, Figure 7B).

### 3.7. Topical Application of VitB6 Alleviated NF-κB-Mediated Inflammation in Cornea of PM2.5-Induced Dry Eye in Mice

**Figure 8:**
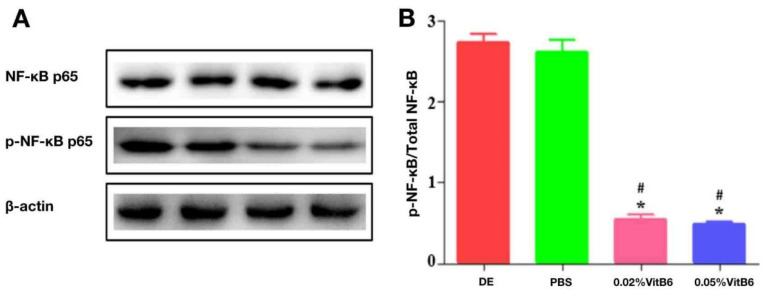
Activation of NF-κB in the corneas of different groups of mice was detected by WB using β-actin as an internal reference. (**A**) WB assay: After 14 days of therapy, mice in the 0.02% VitB6 and 0.05% VitB6 treatment groups had significantly lower levels of p-NF-κB p65 expression in their corneas than mice in the dry eye and PBS treatment groups. (**B**) IOD results are statistically analyzed. The central corneal p-NF-κB/Total NF-κB values were considerably lower after 14 days of treatment than in the PBS-treated group and the dry eye group (n = 10 mice/group). Note: * *p* < 0.05, vs. dry eye, # *p* < 0.05, vs. PBS. Abbreviations: DE, dry eye; PBS, phosphate-buffered saline; VitB6, vitamin B6; WB, Western blot.

The p-NF-κB p65 expression levels in the corneas of mice in the 0.02 percent VitB6 and 0.05 percent VitB6 treatment groups were considerably lower than those in the DE model and PBS treatment groups after 14 days of therapy (F = 538.418, *p* = 0.0001, Figure 8A), according to WB data. IOD results are statistically analyzed. After 14 days of treatment, the central corneal p-NF-κB/Total NF-κB values were significantly lower than in the PBS-treated group and the dry eye group (Figure 8B).

## 4. Discussion

Since the first official definition of DE was published in 1995, it has been considered a disease. In recent years, however, an emerging consensus on DE has been to recognize it as an umbrella term for a range of diseases. Based on this new consensus, the second meeting of the TFOS Dry Eye Workshop II (TFOS DEWS II) of the International Tear Film and Ocular Surface Society in 2017 also redefined DE precisely, namely “DE is a multifactorial disease of the ocular surface characterized by a loss of tear film homeostasis with ocular symptoms in which tear film instability and tear hyperosmolarity, ocular surface inflammation, injury and neurosensory abnormalities all play an etiological role” [24]. DE has a significant negative impact on the physical and mental health of patients, preventing them from carrying out basic daily activities [1]. It is also a highly prevalent, complex inflammatory eye disease with multifactorial involvement, and air pollution is considered to be its main current causative agent [25].

Epidemiological studies of air pollution have shown that human exposure to polluted environments has a significant negative impact on their health [26]. According to the 2018 WHO Global Air Quality Report, approximately 92% of the world’s population live in an environment that is below the air quality guideline (AQG) standards established by the WHO, and approximately 3 million people died prematurely worldwide in 2012 due to health problems caused by air pollution [27]. PM2.5 is a particulate body floating in the air with a diameter of less than 2.5 μm, and it is an important indicator for assessing air pollution. In addition to the well-known respiratory damage, many current studies have confirmed that PM2.5 can also cause damage to other multiple organs and systems, such as accelerated skin aging [28] and increased insulin resistance, leading to diabetes and cardiometabolic diseases [29]. A recent study by Jurewicz J et al. concluded that PM2.5 can even reduce male fertility [30]. Another study by Pritam Saha et al. also reported that exposure of pregnant women to PM2.5 air pollution increased the risk of airway diseases in their offspring [31]. Numerous previous studies have confirmed that PM2.5 contributes to the development of DE [32,33]. In order to confirm whether PM2.5 can cause DE-related symptoms, we first conducted in vitro cellular experiments in which HCECs were treated with different concentrations of PM2.5, and they found that a concentration of 5.0 mg/mL PM2.5 solution had the strongest inhibitory effect on the proliferation of HCECs. Scratching experiments also showed that 5.0 mg/mL PM2.5 solution significantly inhibited the migration ability of HCECs. We found that PM2.5 could impair tear film function and cause structural damage to the ocular surface of mice, and that the tissue changes in the ocular surface of mice caused by topical drops of 5 mg/mL PM2.5 were similar to the clinical manifestations of human DEs. Therefore, we concluded that PM2.5 topical drops in mice caused structural damage to the ocular surface. Therefore, we believe that the PM2.5-induced DE mouse model can stably mimic the pathogenic factors and pathological changes consistent with human DE. In particular, ocular surface inflammation, apoptosis of epithelial cells, squamous metaplasia, and loss of cupped cells in the vaulted conjunctiva are considered to be the most important changes in the developmental mechanism of DE. In addition, the model is of good utility and represents the evaporative subtype in terms of preservatives and can be used in subsequent DE studies.

With the progress of industrialization and changes in the lifestyles of the population, the health of the ocular surface is becoming increasingly important, especially DE. The use of glucocorticoids and non-steroidal anti-inflammatory drugs help to control inflammation and relieve ocular discomfort, but the complications they cause should not be ignored. Immunomodulators and some biological agents are more effective but need to be further evaluated in clinical trials. Surgery has obvious indications and contraindications and is suitable for patients with severe DE; however, usually these cases cannot be reversed. Combined with the current state of air pollution and the increasing incidence of DE, the successful construction of a mouse model of PM2.5-induced dry eye has laid the foundation to search further for a potential, effective drug to treat DE.

VitB6 is extensively involved in metabolic, physiological, and developmental processes. Based on its water solubility and high reactivity upon phosphorylation, it has been considered a co-factor for more than 140 biochemical reactions in cells [34] and is involved in many processes such as protein folding, amino acid synthesis, degradation of intracellular compounds, and the biosynthesis of neurotransmitters [35]. Zhong J et al. reported that VitB6 supplementation avoided PM2.5-induced DNA methylation alterations [36]. Given the pathogenesis of DE and the multiple roles of VitB6, we speculate that it may be a potential agent for DE treatment and could play a role in the treatment of mice models of DE induced by airborne PM2.5 pollution.

For this reason, after successfully establishing the models, we performed VitB6 intervention in the PM2.5 mouse DE model. We found that, compared to the DE model group and the PBS-treated group, mice with different concentrations of VitB6 eye drops showed an increase in tear secretion, prolonged TBUT, reduced corneal staining, and a decrease in inflammation scores. There was less inflammatory cell infiltration. These results suggested that VitB6 can protect the corneal epithelium and maintain the stability of the tear film, reducing the inflammatory response of the ocular surface. However, the mechanism of action of VitB6 in reversing dry eye lesions remains to be elucidated.

DE occurs when the patient is continuously exposed to injurious stimuli or when there is a large activation of injurious receptors due to cellular damage, resulting in a painful response (also known as injurious pain). In addition, the corneal and conjunctival nerves may also become dysfunctional, resulting in abnormal signaling and ultimately neuropathic pain. Therefore, maintaining stable nerve function and promoting functional recovery may alleviate the injurious pain caused by DE, thereby improving clinical symptoms and reducing the physical, psychological, and work and life impact of DE. In addition, studies have shown that nerve damage in DE patients can lead to decreased corneal perception [37], resulting in a decrease in the lacrimal gland’s response to ocular surface stimuli, which can lead to a decrease in reflex tears and ultimately further aggravate ocular surface damage [38]. This role of B vitamins in analgesia and neuroprotection and nutrition has long been reported and clinically used in numerous studies [16,17]. To investigate whether VitB6 mediates DE treatment with the same effect of promoting nerve repair and relieving nerve dysfunction, we observed changes in the nerve fiber density, branching, and curvature in the basal layer of the corneal epithelium. Nerve staining may be non-specific, so we used the most intuitive in vivo confocal microscopy to observe and quantitatively analyze the changes in corneal nerves. We found that the nerve fiber density and branching in the basal layer of the corneal epithelium increased after VitB6 treatment relative to the control group, while nerve curvature scores also improved after 14 days of treatment. This result strongly suggested a possible mechanism by which VitB6 mediates DE treatment, i.e., nourishing and promoting corneal nerve repair. The rebound in tear secretion in mice after 14 days of treatment may be explained by VitB6 repairing ocular surface nerve damage, maintaining nerve function, and promoting recovery of corneal perception.

Loss of cupped cells has been shown to be an important pathological change in the development of DE. In patients with DE, when chronic inflammation or severe inflammation persists, there is a corresponding decrease in the number of cupped cells [39,40]. At the same time, as the stability of the tear film is destabilized during DE pathology, this results in loss of the outer layer of corneal epithelial cells, which eventually triggers massive apoptosis of corneal epithelial cells. Therefore, in order to evaluate the status of cupped cells and the level of epithelial apoptosis, we further performed PAS staining and TUNEL staining at day 14 of the established model, and the results showed that the number of apoptotic cells in the corneas of mice decreased after VitB6 treatment compared with the control group, but the number of cupped cells at the vaulted conjunctiva did not show a statistical difference though it rebounded. Further expansion of the sample size is needed for future follow-up. In combination with the above results, we speculate that the effect of VitB6 on reducing corneal epithelial apoptosis may be complementary to its anti-inflammatory properties.

Inflammation can lead to epithelial apoptosis and squamous epithelial metaplasia [40,41]. Current studies have reported squamous epithelial metaplasia on the ocular surface in the moderate and severe DE stages due to the prolonged presence of chronic inflammation [42]. Squamous epithelial metaplasia is also an important indicator of increased DE and is the most dangerous pathological change that causes vision loss in DEs. K10 is a highly specific and sensitive indicator of the state of pathological keratinization and is now also widely used [43]. In the present experiment, we measured the protein expression level of K10 by immunofluorescence. The results revealed that the protein expression level of K10 was relatively lower in mice treated with VitB6 compared to the control group. This result suggested that blocking squamous metaplasia of the corneal epithelium is another important mechanism by which VitB6 mediates the efficacy of DE treatment. The growth and metabolism of cells and the effects of drugs are fundamentally based on their specific molecular mechanisms. The multi-effect mechanism of action of VitB6 also makes it inevitable that there are many effector molecules. It has been shown that VitB6 attenuates the inflammatory response by modulating the NF-κB pathway [14]. However, no studies have been reported on VitB6-mediated DE treatment. A study on a rabbit dry eye group confirmed that the NF-κB signaling pathway is closely associated with the development of DE and that activation of the NF-κB pathway may be one of the initiating mechanisms of DE [44]. NF-κB is a nuclear transcription factor that consists of five members in mammals, including Rel, RelA (p65), RelB, NF-κB1 (p50), and NF-κB2 (p52). Numerous studies have established that the main functions of NF-κB are cell cycle and proliferation regulation, anti-apoptosis, and secretion of cytokines, all of which play important roles in organismal physiology, inflammation and tumors [45]. Therefore, the question of whether VitB6 exerts an anti-DE effect by activating the NF-κB signaling pathway and causing the transcription of many effector proteins remains. It is also an important research direction for DE treatment.

In the present experiment, we examined the expression levels of p-NF-κB p65 and NF-κB p65 in the corneas of DE mice using a WB technique. The results showed that the expression of p-NF-κB p65 in the VitB6-treated group was significantly lower than that in the control group, indicating that NF-κB was activated. Therefore, we speculate that the mechanism of PM2.5-induced DE by VitB6 treatment may be mediated by the activation of NF-κB. It would be of great value to verify the potential relationship between NF-κB activation and DE progression, which may facilitate the progress of DE studies. VitB6 has antioxidant properties and anti-inflammatory effects that can remove free radicals and reduce oxidative stress damage to eye tissue. At the same time, it can reduce the probability of eye inflammation and reduce inflammatory symptoms. We hypothesized that VitB6 might scour free radicals and reduce oxidative stress damage to eye tissue, potentially reducing the activation of the NF-κB pathway. The anti-inflammatory effect of vitamin B6 may also be achieved by regulating the expression of inflammatory mediators. Although the direct mechanism of action is unknown, VitB6 may reduce ocular inflammation by influencing the expression of inflammatory mediators downstream of the NF-κB pathway, and these could be further explored in our future studies.

After this study, we have gained further insight into VitB6 for the treatment of PM2.5-induced DE, which may be a safe and effective clinical agent with promising applications. It provides a theoretical basis for addressing air pollution-induced ocular surface damage, especially the unexplained neurosensory abnormalities after dry eye formation, and opens up new avenues for current DE treatment.

## 5. Conclusions

In summary, our study confirmed the efficacy of VitB6 eye drops in a PM2.5-induced dry eye group in mice. The application of VitB6 for the treatment of air pollution-induced dry eyes may be a safe and effective clinical agent with promising applications. This study provides new insights for the treatment of DE, and indicates that VitB6 has great potential to be a therapeutic agent in the clinical treatment of DE.

### Limitation

The efficacy of 0.05% VitB6 eye drops was slightly better than 0.02% VitB6 eye drops in this study, but no statistically significant difference was found. Therefore, the optimal therapeutic dose, frequency of use and the exact molecular regulatory mechanism need to be confirmed by further studies. Further investigations are needed to explore the exact mechanism of VitB6 regulating the NF-κB signaling pathway.

## Figures and Tables

**Table 1:** Comparison of SIT and TBUT in four groups of mice before and after treatment.

Groups	SIT (mm)			TBUT (s)		
	0 D	7 D	14 D	O D	7 D	14 D
PM2.5	1.73 ± 0.23	2.35 ± 0.49 ^c^	3.45 ± 1.22 ^c^	2.6 ± 0.97	3.0 ± 1.16	3.3 ± 0.95 ^c^
PBS	1.78 ± 0.21	2.42 ± 0.55 ^c^	3.62 ± 1.43 ^c^	2.7 ± 0.95	2.9 ± 0.88	3.2 ± 0.92 ^c^
0.02% VitB6	1.75 ± 0.29	3.21 ± 0.72 ^ab^	5.05 ± 1.22 ^ab^	2.8 ± 0.79	3.5 ± 1.08	5.8 ± 1.32 ^ab^
0.05% VitB6	1.79 ± 0.33	4.89 ± 1.14 ^abc^	5.84 ± 1.27 ^ab^	2.7 ± 0.95	3.8 ± 1.23	6.1 ± 1.20 ^ab^

Notes: Each value represents the mean ± SD, n = 10. ^a^ *p* < 0.05, vs.PM2.5; ^b^ *p* < 0.05, vs. PBS; ^c^ *p* < 0.05, vs. 0.02% VitB6. Abbreviations: D, Day; PM, particulate matter; PBS, phosphate-buffered saline; VitB6, vitamin B6; SIT, Schirmer I test; TBUT, Tear Break-up time.

**Table 2:** Comparison of inflammation index and FL staining in four groups of mice before and after treatment.

Groups	Inflammation Index			FL		
	0 D	7 D	14 D	0 D	7 D	14 D
PM2.5	0.31 ± 0.12	0.29 ± 0.09 ^c^	0.23 ± 0.05 ^c^	9.5 ± 1.43	9.8 ± 1.40 ^c^	9.6 ± 1.07 ^c^
PBS	0.28 ± 0.15	0.27 ± 0.07 ^c^	0.25 ± 0.06 ^c^	10.9 ± 1.79	10.1 ± 1.45 ^c^	9.8 ± 1.14 ^c^
0.02% VitB6	0.29 ± 0.11	0.11 ± 0.06 ^ab^	0.07 ± 0.03 ^ab^	9.7 ± 1.49	8.5 ± 1.45 ^ab^	7.2 ± 1.55 ^ab^
0.05% VitB6	0.32 ± 0.09	0.06 ± 0.08 ^ab^	0.03 ± 0.01 ^ab^	9.4 ± 1.65	8.4 ± 1.27 ^ab^	7.0 ± 1.25 ^ab^

Notes: Each value represents the mean ± SD, n = 10. ^a^ *p* < 0.05, vs.PM2.5; ^b^ *p* < 0.05, vs. PBS; ^c^ *p* < 0.05, vs. 0.02% VitB6. Abbreviations: D, Day; PM, particulate matter; PBS, phosphate-buffered saline; VitB6, vitamin B6; FL, fluorescein stain.

**Table 3:** Comparison of RB and LG staining in four groups of mice before and after treatment.

Groups	RB			LG		
	0 D	7 D	14 D	0 D	7 D	14 D
PM2.5	5.1 ± 0.74	4.4 ± 1.27 ^c^	4.1 ± 0.99 ^c^	6.3 ± 0.67	5.4 ± 1.07 ^c^	5.2 ± 1.03 ^c^
PBS	4.8 ± 0.79	4.2 ± 1.03 ^c^	4.2 ± 0.92 ^c^	6.5 ± 0.53	5.2 ± 1.03 ^c^	5.0 ± 1.05 ^c^
0.02% VitB6	5.2 ± 0.63	3.3 ± 1.16 ^a^	3.2 ± 1.03 ^ab^	6.2 ± 0.79	4.1 ± 1.11 ^ab^	3.9 ± 0.99 ^ab^
0.05% VitB6	4.9 ± 0.74	3.2 ± 0.92 ^ab^	3.1 ± 0.89 ^ab^	6.4 ± 0.70	4.0 ± 1.16 ^ab^	3.6 ± 1.27 ^ab^

Notes: Each value represents the mean ± SD, n = 10; ^a^ *p* < 0.05, vs. PM2.5; ^b^ *p* < 0.05, vs. PBS; ^c^ *p* < 0.05, vs. 0.02% VitB6. Abbreviations: D, Day; PM, particulate matter; PBS, phosphate-buffered saline; VitB6, vitamin B6; RB, Rose bengal staining; LG, Lissamine Green staining.

**Table 4:** Comparison of nerve fiber density under the basement membrane of corneal epithelium before and after treatment in four groups of mice.

Groups	Nerve Fiber Density (mm/mm^2^)		
	0 D	7 D	14 D
PM2.5	12.67 ± 1.52	12.80 ± 1.23 ^c^	12.87 ± 1.50 ^c^
PBS	12.54 ± 1.47	12.65 ± 1.28 ^c^	12.90 ± 1.31 ^c^
0.02% VitB6	12.69 ± 1.28	13.92 ± 0.97 ^ab^	14.52 ± 1.37 ^ab^
0.05% VitB6	12.72 ± 1.61	13.99 ± 1.12 ^ab^	14.87 ± 1.21 ^ab^

Notes: Each value represents the mean ± SD, n = 10. ^a^ *p* < 0.05, vs.PM2.5; ^b^ *p* < 0.05, vs. PBS; ^c^ *p* < 0.05, vs. 0.02% VitB6. Abbreviations: D, Day; PM, particulate matter; PBS, phosphate-buffered saline; VitB6, vitamin B6.

**Table 5:** Comparison of the number of nerve fiber branches under the basement membrane of corneal epithelium before and after treatment in four groups of mice.

Groups	Fiber Branching (Number of Branches/Figure)		
	0 D	7 D	14 D
PM2.5	5.15 ± 0.63	5.15 ± 0.41 ^c^	5.10 ± 0.46 ^c^
PBS	5.05 ± 0.44	5.15 ± 0.53 ^c^	5.20 ± 0.54 ^c^
0.02% VitB6	5.25 ± 0.49	5.55 ± 0.60 ^ab^	5.95 ± 0.64 ^ab^
0.05% VitB6	5.30 ± 0.48	5.75 ± 0.54 ^ab^	6.10 ± 0.57 ^ab^

Notes: Each value represents the mean ± SD, n = 10. ^a^ *p* < 0.05, vs.PM2.5; ^b^ *p* < 0.05, vs. PBS; ^c^ *p* < 0.05, vs. 0.02% VitB6. Abbreviations: D, Day; PM, particulate matter; PBS, phosphate-buffered saline; VitB6, vitamin B6.

**Table 6:** Comparison of subbasal nerve fiber curvature scores of corneal epithelia in four groups of mice before and after treatment.

Groups	Nerve Fiber Curvature Score		
	0 D	7 D	14 D
PM2.5	2.33 ± 0.35	2.35 ± 0.40	2.38 ± 0.32 ^c^
PBS	2.36 ± 0.41	2.36 ± 0.41	2.40 ± 0.34 ^c^
0.02% VitB6	2.38 ± 0.38	2.27 ± 0.35	2.06 ± 0.31 ^ab^
0.05% VitB6	2.35 ± 0.33	2.14 ± 0.36	1.96 ± 0.37 ^ab^

Notes: Each value represents the mean ± SD, n = 10. ^a^ *p* < 0.05, vs. PM2.5; ^b^ *p* < 0.05, vs. PBS; ^c^ *p* < 0.05, vs. 0.02% VitB6. Abbreviations: D, Day; PM, particulate matter; PBS, phosphate-buffered saline; VitB6, vitamin B6.

## Data Availability

The datasets used and/or analyzed during the present study are available from the corresponding author on reasonable request.
